# Echocardiographic Assessment of Biventricular Mechanics of Fetuses and Infants of Gestational Diabetic Mothers: A Systematic Review and Meta-Analysis

**DOI:** 10.3390/children11121451

**Published:** 2024-11-28

**Authors:** Andrea Sonaglioni, Antonino Bruno, Gian Luigi Nicolosi, Stefano Bianchi, Michele Lombardo, Paola Muti

**Affiliations:** 1 Division of Cardiology, IRCCS MultiMedica, 20123 Milan, Italy; michele.lombardo@multimedica.it; 2 Laboratory of Innate Immunity, IRCCS MultiMedica, 20138 Milan, Italy; antonino.bruno@multimedica.it; 3 Laboratory of Immunology and General Pathology, Department of Biotechnology and Life Sciences, University of Insubria, 21100 Varese, Italy; 4 Division of Cardiology, Policlinico San Giorgio, 33170 Pordenone, Italy; gianluigi.nicolosi@gmail.com; 5 Division of Gynecology and Obstetrics, IRCCS MultiMedica, 20123 Milan, Italy; stefano.bianchi@unimi.it; 6 Department of Biomedical, Surgical and Dental Sciences, University of Milan, 20122 Milan, Italy; 7 IRCCS MultiMedica, 20138 Milan, Italy

**Keywords:** fetuses, infants, gestational diabetes mellitus, biventricular, global longitudinal strain

## Abstract

Background: Gestational diabetes mellitus (GDM) is the most common complication in pregnancy, representing a serious risk for the mother and fetus. Identifying new biomarkers to ameliorate the screening and improving GDM diagnosis and treatment is crucial. During the last decade, a few studies have used speckle tracking echocardiography (STE) for assessing the myocardial deformation properties of fetuses (FGDM) and infants (IGDM) of GDM women, providing not univocal results. Accordingly, we performed a meta-analysis to examine the overall influence of GDM on left ventricular (LV) and right ventricular (RV) global longitudinal strain (GLS) in both FGDM and IGDM. Methods: All echocardiographic studies assessing conventional echoDoppler parameters and biventricular strain indices in FGDM and IGDM vs. infants born to healthy pregnant women, selected from PubMed and EMBASE databases, were included. The studies performed on FGDM and IGDM were separately analyzed. The subtotal and overall standardized mean differences (SMDs) in LV-GLS and RV-GLS in FGDM and IGDM studies were calculated using the random-effect model. Results: The full texts of 18 studies with 1046 babies (72.5% fetuses) born to GDM women and 1573 babies of women with uncomplicated pregnancy (84.5% fetuses) were analyzed. Compared to controls, FGDM/IGDM were found with a significant reduction in both LV-GLS [average value −18.8% (range −11.6, −24.2%) vs. −21.5% (range −11.8, −28%), *p* < 0.05)] and RV-GLS [average value −19.7% (range −13.7, −26.6%) vs. −22.4% (range −15.5, −32.6%), *p* <0.05)]. Large SMDs were obtained for both LV-GLS and RV-GLS studies, with an overall SMD of −0.91 (95%CI −1.23, −0.60, *p* < 0.001) and −0.82 (95%CI −1.13, −0.51, *p* < 0.001), respectively. Substantial heterogeneity was detected for both LV-GLS and RV-GLS studies, with an overall I^2^ statistic value of 92.0% and 89.3%, respectively (both *p* < 0.001). Egger’s test gave a *p*-value of 0.10 for LV-GLS studies and 0.78 for RV-GLS studies, indicating no publication bias. In the meta-regression analysis, none of the moderators (gestational age, maternal age, maternal body mass index, maternal glycosylated hemoglobin, white ethnicity, GDM criteria, ultrasound system, frame rate, FGDM/IGDM heart rate, and anti-diabetic treatment) were significantly associated with effect modification in both groups of studies (all *p* > 0.05). The sensitivity analysis supported the robustness of the results. Conclusions: GDM is independently associated with biventricular strain impairment in fetuses and infants of gestational diabetic mothers. STE analysis may allow for the early detection of subclinical myocardial dysfunction in FGDM/IGDM.

## 1. Introduction

Gestational diabetes mellitus (GDM) is defined as any glucose intolerance with the onset or first recognition during pregnancy [1]. Its global prevalence, based on the International Association of Diabetes and Pregnancy Study Groups (IADPSG) criteria, is approximately 15% [2]. This condition significantly affects not only pregnant women with GDM but also fetuses (FGDM) and infants (IGDM) of gestational diabetic mothers.

GDM women have an increased risk of hypertension and, consequently, preeclampsia or eclampsia during pregnancy and of developing type 2 diabetes and cardiovascular diseases later in life [3,4].

Infants born to gestational diabetic mothers have an increased risk of neonatal complications, such as macrosomia, neonatal hypoglycemia, hyperbilirubinemia, shoulder dystocia, birth trauma, and stillbirth [5].

GDM has a negative impact on the structure and function of the fetal heart and fetal–placental circulation, causing fetal hypertrophic cardiomyopathy [6], more evident in the third trimester of pregnancy. FGDM and IGDM cardiomyopathy have been traditionally studied with M-mode, two-dimensional (2D) transthoracic echocardiography (TTE), and pulsed wave (PW) tissue Doppler imaging (TDI), demonstrating asymmetric myocardial hypertrophy, affecting mainly the interventricular septum, commonly associated with diastolic dysfunction, preceding systolic dysfunction, and in some cases associated with progression to symptomatic heart failure [7]. However, fetuses or infants of GDM mothers may develop a subclinical decrease in systolic and diastolic myocardial function even in the absence of symptoms and of cardiac hypertrophy [8]. GDM early detection, diagnosis, and therapies can improve the prognosis and the health of both mother and fetus and reduce social costs.

Recent advances in echocardiographic imaging have led to the introduction of speckle tracking echocardiography (STE), a more sensitive diagnostic tool to detect subclinical abnormalities of fetal cardiac function [9].

To date, a few studies have used STE analysis for assessing myocardial deformation properties of fetuses and infants of women affected by GDM, providing not univocal results. The majority of these studies evaluated both left ventricular (LV) and right ventricular (RV) mechanics by using traditional 2D-TTE implemented with STE.

The present systematic review and meta-analysis have been designed to summarize the main findings of all studies that examined both LV and RV myocardial deformation indices obtained by STE analysis in fetuses and infants of GDM women in comparison to those measured in fetuses and infants of healthy pregnant women. This review will also provide an overview of the main pathophysiological mechanisms underpinning GDM-related subclinical myocardial dysfunction in FGDM and IGDM.

## 2. Materials and Methods

This systematic review and meta-analysis were developed according to the Preferred Reporting Items for Systematic Reviews and Meta-analyses (PRISMA) guidelines [10], and was registered in PROSPERO (CRD42024508558).

### 2.1. Search Strategy

A comprehensive search of all articles assessing conventional echoDoppler parameters and biventricular strain indices in fetuses and infants born to women with GDM, regardless of the time frame, was carried out by two independent reviewers (A.S. and M.L.) through January 2024 using Medline and EMBASE databases. The search strategy included the following terms: “fetuses” OR “infants” AND “gestational diabetes mellitus” OR “GDM” AND “cardiac function” AND “subclinical myocardial dysfunction” AND “left ventricular mechanics” OR “left ventricular global longitudinal strain” OR “LV-GLS” AND “right ventricular mechanics” OR “right ventricular global longitudinal strain” OR “RV-GLS”.

### 2.2. Eligibility Criteria

Echocardiographic studies that evaluated conventional echoDoppler parameters and biventricular strain indices in fetuses and infants born to women with GDM in comparison to fetuses and infants of healthy women were included. Conversely, non-echocardiographic studies, echocardiographic studies without STE-derived assessment of LV global longitudinal strain (GLS) and/or RV-GLS, studies conducted in fetuses and infants of GDM women without control groups, studies performed on fetuses and infants of mothers with pre-gestational diabetes, studies performed on fetuses and infants of mothers with hypertensive disorders of pregnancy, published documents different from research articles, and finally, non-English language articles were excluded.

### 2.3. Study Selection and Data Extraction

Two independent reviewers (A.S. and M.L.) screened the records in accordance with the eligibility criteria for this review. Information concerning the following was independently collected by two reviewers: (1) maternal age, self-reported ethnicity, body mass index (BMI), and glycosylated hemoglobin (HbA1C); (2) GDM diagnostic criteria; (3) the gestational age of FGDM at echocardiographic assessment; (4) the estimated fetal weight (EFW) of FGDM and the average birth weight of IGDM; (5) the heart rate of FGDM/IGDM at echocardiographic assessment; (6) the ultrasound system used for STE analysis; (7) echocardiographic parameters measured by 2D-TTE and 2D-STE; (8) follow-up data, if any. Possible discrepancies between the reviewers were resolved through a consensus discussion with the involvement of a third author (G.L.N.).

### 2.4. Risk-of-Bias Assessment

The risk of bias (RoB) in the included studies was assessed by using the National Institutes of Health (NIH) Quality Assessment of Case-Control Studies [11]. The investigators assigned an overall “good”, “fair”, or “poor” rating to each study. The quality rating was independently estimated by two authors (A.S. and G.L.N.). Disagreement was resolved by consensus.

**Figure 1:**
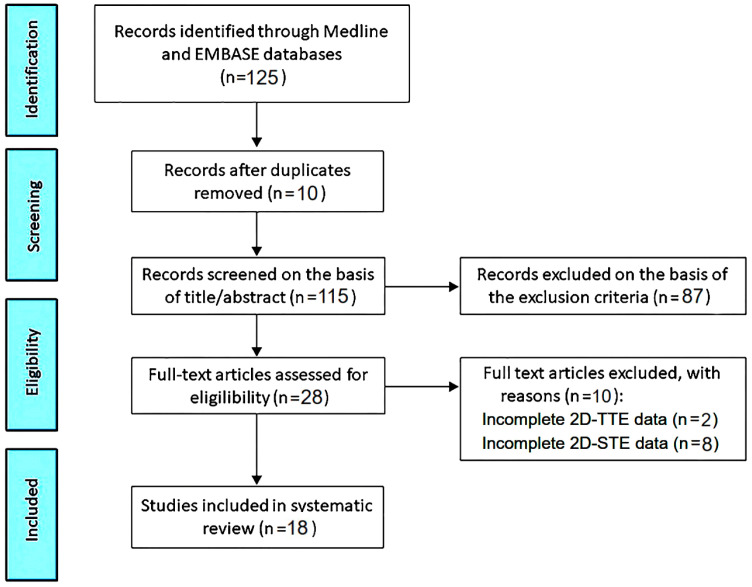
Flow diagram used for identifying the included studies. Note—2D, two-dimensional; STE, speckle tracking echocardiography; TTE, transthoracic echocardiography.

Figure 1 illustrates the PRISMA flow diagram used for identifying the included studies.

### 2.5. Statistical Analysis

The effect of GDM on biventricular mechanics in FGDM and IGDM was quantified. Continuous data (LV-GLS and RV-GLS) were pooled as the standardized mean difference (SMD) comparing the FGDM/IGDM groups with healthy controls. The studies performed on FGDM and IGDM were separately analyzed. The subtotal and overall standardized mean differences (SMDs) in LV-GLS and RV-GLS were calculated using the random-effect model due to the high statistical heterogeneity among the included studies. The I-squared statistic (I^2^) was used to quantify the between-study heterogeneity. Begg’s funnel plots and Egger’s test were used to test any publication bias. Meta-regression was performed to determine if there was an effect modification on the association between GDM and both LV-GLS and RV-GLS in FGDM/IGDM by several moderators, such as gestational age at echocardiographic assessment, maternal age, maternal BMI, maternal HbA1C, white ethnicity, GDM criteria, ultrasound system used for strain echocardiographic imaging, the frame rates used during echocardiographic examination, FGDM/IGDM heart rate, and the anti-diabetic treatment. Finally, a sensitivity analysis was performed by investigating the effect of individual studies on the overall meta-analysis. The 95% confidence intervals (CIs) were calculated and two-tailed *p* values less than 0.05 were considered to be statistically significant. Statistical analysis was performed by using Comprehensive Meta-Analysis version 3.0 (Biostat, Englewood, NJ, USA).

## 3. Results

### 3.1. Clinical Findings

The initial search yielded a total of 125 studies. Of those, 10 (8%) were removed as duplicates. After screening the titles and abstracts, a further 87 studies (69.6%) were removed based on the exclusion criteria. Assessing the full text of the remaining 28 studies (22.4%) resulted in a further 10 exclusions (8%). A total of 18 studies (14.4%) [12,13,14,15,16,17,18,19,20,21,22,23,24,25,26,27,28,29] were thus included in this systematic review, totaling 1046 babies born to gestational diabetic mothers (72.5% fetuses and 27.5% infants) and 1573 babies of women with uncomplicated pregnancy (84.5% fetuses and 15.5% infants).

The main findings of the 18 studies included in the present systematic review and meta-analysis are summarized in Table 1.

The included studies were published between 2012 and 2023. Five studies were performed in China, two in the USA, two in the United Kingdom, and one in Egypt, Japan, Ireland, India, Italy, Portugal, Germany, Australia, and Romania. Self-reported ethnicity was White in ten studies, Asian in seven studies, and Black in one study. Twelve studies (66.7% of the total) examined FGDM, whereas the remaining six studies (33.3% of the total) evaluated cardiac mechanics in IGDM in the perinatal period. The average gestational age of FGDM at echocardiographic assessment was 31.6 weeks (range 25.3–38.2 weeks). The vast majority of FGDM studies (91.7% of the total) performed echocardiographic examination of FGDM during the third trimester of pregnancy, whereas only one study [14] was conducted during the second trimester.

Mean maternal age was 32.1 yrs (range 28.4–34.1 yrs), whereas the mean maternal BMI was 29.9 kg/m^2^ (range 24.5–45.4 kg/m^2^). The mean HbA1C was 5.7% (range 5.3–6.4%). Regarding GDM diagnostic criteria, the vast majority of studies (83.3% of the total) used the oral glucose tolerance test (OGTT) method, two studies used the IADPSG criteria, and only one study used the American Diabetes Association (ADA) criteria. All studies conducted on FGDM used the OGTT method.

The average EFW of FGDM was 2103 g (range 1048–3530 g), whereas the average birth weight of IGDM was 3386 g (range 2911–3600 g). The EFW was greater in FGDM/IGDM than controls in five studies (27.8% of the total), whereas it was similar in both FGDM/IGDM and controls in eight studies (72.2% of the total). The percentage of FGDM/IGDM with macrosomia ranged between 18.2% and 24.2%. The average heart rate at echocardiographic assessment was 141 bpm (range 137–147 bpm) in FGDM and 143 bpm (range 125–158 bpm) in IGDM.

With regards to STE analysis, twelve studies (66.6% of the total) evaluated biventricular mechanics by using a General Electric (GE) ultrasound machine, three studies by Philips software, two studies by Siemens software, and one study by Canon software. The average frame rate used for strain analysis was 113 (range 50–182) frames per second (fps).

A total of 88.8% of studies examined FGDM during pregnancy and IGDM in the perinatal period without providing follow-up data, whereas only two studies performed an echocardiographic re-evaluation in the postpartum period, at 6 weeks and 6 months [28] and at 2 months [29] after delivery, respectively.

### 3.2. Transthoracic Echocardiography Findings

Main conventional echocardiographic parameters assessed in both FGDM/IGDM and controls included the following: (1) interventricular septum (IVS) thickness, LV end-diastolic diameter (EDD), relative wall thickness (RWT), and left ventricular mass index (LVMi, calculated by Devereux’s formula) as indices of LV geometry; (2) the E/A ratio and the E/e’ ratio as indices of LV diastolic function and LV filling pressures, respectively; (3) LV-fractional shortening (FS), LV-fractional area change (FAC), left ventricular ejection fraction (LVEF, estimated with the biplane modified Simpson’s method), LV-stroke volume (SV), mitral annular plane systolic excursion (MAPSE), and LV-myocardial performance index (MPI) as indices of LV systolic function; (4) EDD-right ventricular inflow tract (RVIT) as an index of RV size; (5) finally, tricuspid annular plane systolic excursion (TAPSE), RV-Tei index, and RV-FAC as indices of RV systolic function.

Table 2 summarizes all conventional and functional echocardiographic parameters measured in FGDM/IGDM and controls by the included studies.

The most commonly assessed echocardiographic indices were LVEF (calculated in 55.5% of studies), IVS thickness (measured in half of studies), and E/A ratio (estimated in 38.8% of studies). The remaining echocardiographic indices were determined in a reduced number of studies ranging from 11.1% to 27.7% of the total.

Analysis of LV morphology and structure revealed that, compared to controls, FGDM/IGDM were found with significantly greater IVS thickness, RWT, and LVMi.

Evaluation of LV diastolic function showed that the E/A ratio was significantly lower in FGDM/IGDM than controls, whereas the E/e’ ratio was significantly higher in FGDM/IGDM than controls, averaging 10.4 vs. 7.9.

Even if preserved, the magnitude of the main indices of LV systolic function (LV-FAC, LVEF, LV-SV, and MAPSE) was significantly lower in FGDM/IGDM than controls (all *p* <0.05). The remaining indices of LV systolic function, i.e., LV-FS and LV-MPI, were not statistically different in the two groups of babies.

Concerning RV size and systolic function, EDD-RVIT was similar in both FGDM/IGDM and controls, whereas TAPSE, RV-Tei index, and RV-FAC were significantly lower in FGDM/IGDM than controls.

### 3.3. Echocardiographic Deformation Imaging Findings

LV-GLS was assessed by all included studies. Despite normal LV size, LV-GLS was significantly lower in FGDM/IGDM in comparison to controls (average value −18.8% vs. −21.5%, *p* < 0.05) and slightly reduced in comparison to the accepted reference values [30]. However, the LV-GLS magnitude was not statistically different between FGDM/IGDM and controls in six studies (33.3% of the total).

**Figure 2:**
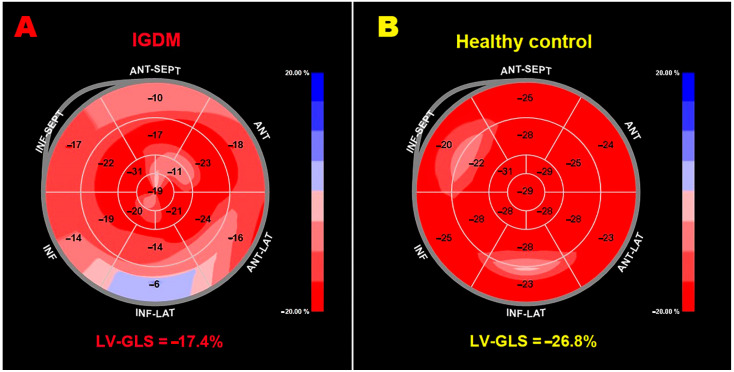
Representative examples of LV-GLS bull’s eye plot assessed by 2D-STE analysis in the perinatal period in an infant born to mother with GDM (**A**) and in an infant born to mother with uncomplicated pregnancy (**B**). Note—2D, two-dimensional; GDM, gestational diabetes mellitus; GLS, global longitudinal strain; IGDM, infant born to gestational diabetic mother; LV, left ventricular; STE, speckle tracking echocardiography.

Representative examples of LV-GLS bull’s eye plots obtained in the perinatal period in an IGDM and in a healthy control are depicted in Figure 2A,B, respectively.

LV global circumferential strain (GCS) was measured in only three studies [14,26,29] (16.6% of the total). Two of these studies [14,29] demonstrated a significant impairment in LV-GCS in FGDM/IGDM vs. controls, whereas Iwashima S. et al. [26] found that the endocardial GCS magnitude was significantly higher in IGDM than controls.

RV strain was assessed in 14 studies (77.7% of the total), totaling 871 babies of gestational diabetic mothers and 1329 babies of healthy pregnant women. The RV-GLS magnitude was significantly lower in FGDM/IGDM than controls (average value −19.7% vs. −22.4%, *p* < 0.05). All of these studies found a significantly lower RV strain magnitude in FGDM/IGDM vs. controls, except for Gireadă R. et al. [22] who did not observe any statistically significant differences in RV strain between the two groups of babies.

Regarding anti-diabetic treatment, the percentage of GDM women prescribed with insulin and/or metformin in the included studies ranged from 6.1 to 61% of the total.

Concerning postpartum data, Samanth J. et al. [28] demonstrated a persistent impairment in LV-GLS in IGDM 6 months after delivery. This finding was ascribed by the authors to poor glycemic control during pregnancy. Similarly, our study group [29] found that approximately one-third of IGDM maintained a significant LV-GLS impairment at two months after birth; logistic regression analysis revealed that maternal obesity and uncontrolled diabetes were independently associated with persistent LV subclinical myocardial dysfunction in IGDM over a short-term follow-up.

### 3.4. Risk of Bias Assessment

The NIH quality rating was estimated as good for five studies and fair for thirteen studies. Cohen’s Kappa coefficient indicated a substantial agreement between the reviewers in the RoB assessment, κ = 0.79.

### 3.5. Influence of GDM on LV-GLS in FGDM/IGDM

**Figure 3:**
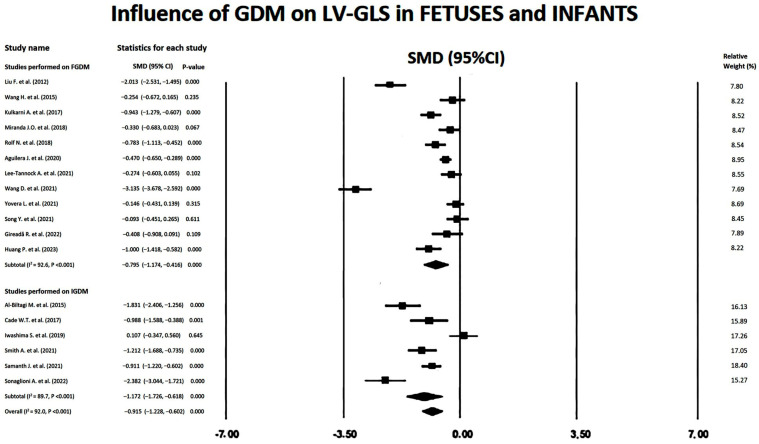
Forest plots showing the influence of GDM on LV-GLS in the included studies [12,13,14,15,16,17,18,19,20,21,22,23,24,25,26,27,28,29]. GDM, gestational diabetes mellitus; GLS, global longitudinal strain; LV, left ventricular.

Forest plots showing the influence of GDM on LV-GLS in FGDM and IGDM are depicted in Figure 3.

**Figure 4:**
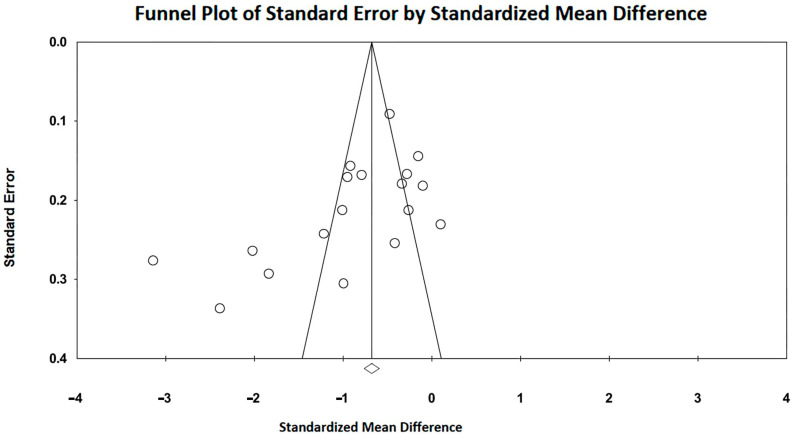
Begg’s funnel plot for the detection of publication bias in LV-GLS studies. GLS, global longitudinal strain; LV, left ventricular.

Large SMDs were obtained for the included studies, with an overall SMD of −0.91 (95%CI −1.23, −0.60, *p* < 0.001). Subgroup analysis showed a larger subtotal SMD for IGDM studies (−1.17; 95%CI −1.73, −0.62, *p* < 0.001) than that obtained for FGDM studies (−0.79, 95%CI −1.17, −0.42). Substantial heterogeneity was detected for the studies that evaluated the influence of GDM on LV-GLS in FGDM/IGDM, with an overall I^2^ statistic value of 92.0% (*p* < 0.001). Egger’s test gave a *p*-value of 0.10, indicating no publication bias. Begg’s funnel plot is illustrated in Figure 4.

In the meta-regression analysis, none of the moderators were significantly associated with an effect modification on the association between GDM and LV-GLS in FGDM/IGDM (all *p* > 0.05) (Table 3).

The sensitivity analysis supported the robustness of the results. Omitting each study sequentially caused a modest variability in SMD from −0.902 (95%CI −1.213, −0.591, *p* < 0.001) to −1.414 (95%CI −2.240, −0.588, *p* = 0.001).

### 3.6. Influence of GDM on RV-GLS in FGDM/IGDM

**Figure 5:**
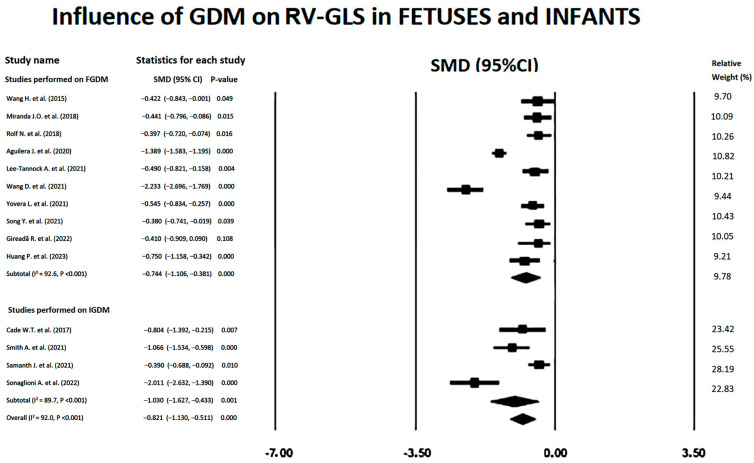
Forest plots showing the influence of GDM on RV-GLS in the included studies [13,15,16,17,18,19,20,21,22,23,25,27,28,29]. GDM, gestational diabetes mellitus; GLS, global longitudinal strain; RV, right ventricular.

Forest plots showing the influence of GDM on RV-GLS in FGDM and IGDM are illustrated in Figure 5.

**Figure 6:**
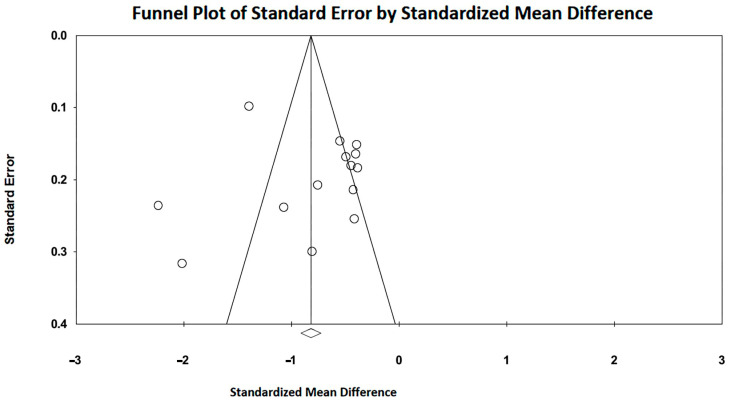
Begg’s funnel plot for the detection of publication bias in RV-GLS studies. GLS, global longitudinal strain; RV, right ventricular.

Large SMDs were obtained for the included studies, with an overall SMD of −0.82 (95%CI −1.13, −0.51, *p* < 0.001). Subgroup analysis showed a larger subtotal SMD for IGDM studies (−1.03; 95%CI −1.63, −0.43, *p* = 0.001) than that obtained for FGDM studies (−0.74, 95%CI −1.11, −0.38). Substantial heterogeneity was detected for the studies that evaluated the influence of GDM on RV-GLS in FGDM/IGDM, with an overall I^2^ statistic value of 89.3% (*p* < 0.001). Egger’s test gave a *p*-value of 0.78, indicating no publication bias. Begg’s funnel plot is illustrated in Figure 6.

In the meta-regression analysis, none of the moderators were significantly associated with an effect modification on the association between GDM and RV-GLS in FGDM/IGDM (all *p* > 0.05) (Table 4).

The sensitivity analysis supported the robustness of the results. Omitting each study sequentially caused a modest variability in SMD from −0.889 (95%CI −1.426, −0.353, *p* = 0.001) to −0.640 (95%CI −1.133, −0.147, *p* = 0.01).

## 4. Discussion

### 4.1. Main Findings of the Systematic Review and Meta-Analysis

This meta-analysis revealed that GDM was independently associated with biventricular GLS impairment in both fetuses and infants of gestational diabetic mothers. We detected a moderate effect size for FGDM studies and a large effect size for IGDM studies.

The effect of GDM on RV and LV mechanics of FGDM and IGDM was not modified by maternal or fetal characteristics.

The attenuation of biventricular longitudinal strain detected by STE was accompanied by a peculiar cardiac remodeling, characterized by LV hypertrophy and LV diastolic dysfunction, in the presence of preserved conventional indices of biventricular systolic function.

Overall, the degree of deterioration of all main echocardiographic indices of biventricular systolic function was mild and similar for both traditional and strain echocardiographic parameters, suggesting that subtle changes in the biventricular longitudinal strain indices may be accompanied by small changes in both LVEF and TAPSE; however, the reduction in LVEF and TAPSE appears less evident, likely due to the wider range of normality for LVEF (≥55%) and TAPSE (≥20 mm) in comparison to that accepted for LV-GLS and RV-GLS (more negative than −20%).

In addition, the subclinical myocardial dysfunction was found to be persistent up to six months postpartum in GDM women with obesity and uncontrolled diabetes.

Finally, all included studies confirmed the high clinical feasibility and reproducibility of both LV- and RV-GLS measurements by 2D-STE in FGDM and IGDM, as previously reported [31,32].

### 4.2. Pathophysiological Mechanisms Underpinning Biventricular GLS Impairment in FGDM/IGDM

**Figure 7:**
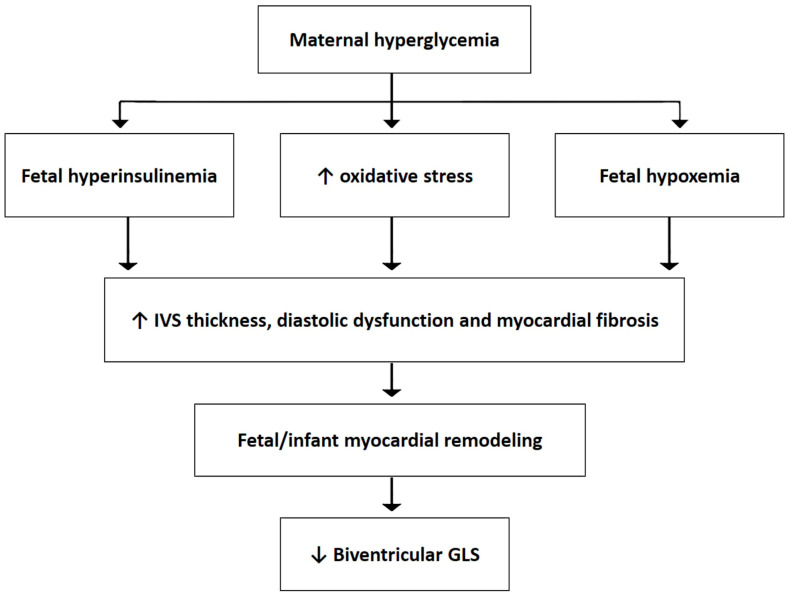
Pathophysiologic mechanisms underpinning biventricular GLS impairment in fetuses and infants of gestational diabetic mothers. GLS, global longitudinal strain; IVS, interventricular septum.

From a pathophysiological point of view, maternal hyperglycemia may induce the impairment of biventricular longitudinal strain in fetuses and infants through a number of mechanisms, summarized in Figure 7.

It is known that maternal hyperglycemia during pregnancy plays a key role in triggering fetal hyperinsulinemia with the development of myocardial wall thickness [33], and cardiac remodeling in both FGDM and IGDM [34].Ventricular hypertrophy, which is commonly more evident at the level of the IVS, determines LV diastolic abnormalities [35]. At the microscopic level, fetal myocardial remodeling is caused by cardiomyocyte hyperplasia and hypertrophy and is characterized by disorganization of the normal pattern of the myofibrils [36]. A hyperglycemic environment may also alter fetal and infant lipid metabolism and cause excessive production of reactive oxygen species (ROS), leading to increased oxidative stress [37] and fetal hypoxemia [38], with consequent myocardial cell damage and accumulation of collagen in the myocardium [39]. Given that longitudinal strain is primarily related to endocardial layer function and integrity, in the presence of hypoxia, the endocardium is the first to be damaged, with a consequent reduction in GLS magnitude. In addition, a number of placental genes related to chronic stress and inflammatory pathways may be associated with increased myocardial vulnerability to stress and reduced cardiac performance in FGDM/IGDM [40]. Myocardial dysfunction in IGDM has also been ascribed to an altered maternal lipid metabolism [41]. Due to the concomitant reduction in myocardial deformation in longitudinal, circumferential, and radial directions detected in FGDM, Kulkarni et al. [14] have hypothesized a myopathic mechanism rather than an adaptive one, as described for cardiomyopathies [42]. Finally, Rolf N. et al. [16] found increased inter- and intra-ventricular dyssynchrony in both LV and RV chambers in FGDM as an effect of maternal hyperglycemia.

With regards to RV mechanics, the included studies revealed a prevalent early impairment in RV GLS, particularly evident in FGDM. This finding was likely related to the important role exerted by the right ventricle during fetal life [43] and to the RV architecture, characterized by a larger midsection diameter and reduced thickness of the lateral wall, making this chamber more susceptible to afterload increase [44]. Moreover, the lower RV performance detected in FGDM/IGDM may be the result of increased RV afterload secondary to LV diastolic dysfunction, leading to increased left atrial pressure and increased pulmonary vascular resistance. RV myocardial dysfunction in FGDM/IGDM has also been ascribed to insulin resistance and amplified sympathetic nervous and renin–angiotensin–aldosterone systems [45]. Finally, as suggested by Tadic M. et al. [46], the RV-GLS magnitude is strongly associated to LV mechanics due to the “interventricular dependence”, given that the RV-GLS magnitude—obtained from the apical four-chamber view—includes not only the RV free wall but also the septal wall.

It is noteworthy that LV- and/or RV-GLS values in FGDM/IGDM may be impaired due to several technical issues, such as (1) the operator’s experience; (2) the need for high-quality 2D images [47]; (3) strict dependence on the frame rate, as a too-low frame rate could result in poor tracking [48]; with this in regard, it is recommended to perform strain analysis with high frame rates (≥80 frames per second) because of the relatively high fetal heart rate [49]; (4) the inter-vendor variability [50]; (5) the dependency on the insonation angle, given that in the apex up/down view, the myocardium is thinly depicted, whereas in the apex perpendicular view, the ventricular septum and papillary muscles are better visualized [51]; (6) finally, the potential influence exerted by a concave-shaped chest conformation on the deformation magnitude of basal myocardial segments, as demonstrated by our study group in infants with pectus excavatum [52]. However, the meta-regression analyses we performed excluded technical factors, such as the ultrasound system employed for speckle tracking analysis, the frame rate used during echocardiographic examination, and the FGDM/IGDM heart rate, which might have a preponderant role in reducing the biventricular GLS magnitude.

### 4.3. Implications for Clinical Practice

Considering the structural remodeling associated with subclinical impairment in biventricular myocardial strain parameters, highlighted by the included studies in both FGDM and IGDM, 2D-TTE implemented with STE analysis may allow clinicians to obtain a more comprehensive cardiological evaluation of these individuals. Indeed, this innovative methodology may reveal a subclinical deterioration of biventricular mechanics in the presence of preserved traditional indices of biventricular systolic function, i.e., LVEF and TAPSE. The use of deformation analysis at the bedside can facilitate more accurate monitoring of cardiac function, especially in fetuses and infants with clinical symptoms.

Given the negative effect of maternal hyperglycemia on fetal and infant cardiac inotropism, an early aggressive maternal glucose and lipid metabolism management should be mandatory in GDM women to attenuate fetal cardiac remodeling and prevent infant myocardial dysfunction during the first months of life. An adequate glycaemic control and weight loss intervention (diet and increased physical activity) in GDM women may reduce the risk of neonatal complications and early metabolic and/or cardiac dysfunction in childhood, youth, and adulthood.

### 4.4. Limitations

The present systematic review included a small number of heterogeneous studies with limited sample sizes. Moreover, studies conducted on fetuses and infants of mothers with pre-gestational diabetes were excluded. Therefore, it is not possible to ascertain whether fetal cardiac remodeling occurs earlier in pregnancy and if it is progressive with advancing gestational age. In addition, the vast majority of studies did not perform a postpartum echocardiographic re-evaluation of IGDM, and in the only two studies that provided postpartum data, the follow-up period was relatively short, with small distances between measurement points of strain echocardiographic indices. Finally, all studies were primarily designed to assess conventional and functional echocardiographic parameters in both FGDM and IGDM and did not evaluate the correlation between maternal Homeostatic Model Assessment for Insulin Resistance (HOMA-IR) and cardiac mechanics obtained in fetuses and infants.

## 5. Conclusions

GDM is independently associated with biventricular longitudinal myocardial strain impairment in both fetuses and infants of gestational diabetic mothers.

STE analysis may allow clinicians to early identify subclinical myocardial dysfunction in fetuses and infants of GDM women.

Further prospective studies are needed to evaluate if more aggressive glycemic control in GDM women during pregnancy could prevent or attenuate such alterations in both FGDM and IGDM.

## Figures and Tables

**Table 1:** Summary of included studies. ADA, American Diabetes Association; FAC, fractional area change; FGDM, fetuses of gestational diabetic mothers; FS, fractional shortening; FWLS, free wall longitudinal strain; GCS, global circumferential strain; GLS, global longitudinal strain; IADPSG, International Association of Diabetes and Pregnancy Study Groups; IGDM, infants of gestational diabetic mothers; IVS, interventricular septum; LV, left ventricular; LVEF, left ventricular ejection fraction; MAPSE, mitral annular plane systolic excursion; MPI, myocardial performance index; NR, not reported; OGTT, oral glucose tolerance test; RV, right ventricular; RWT, relative wall thickness; TAPSE, tricuspid annular plane systolic excursion. The symbol ↑ indicates statistically significant increase of the single parameter in FGDM/IGDM vs. Controls (*p* value < 0.05); the symbol ↔ indicates the absence of statistically significant difference in the single parameter between FGDM/IGDM and Controls (*p* value > 0.05); the symbol ↓ indicates statistically significant reduction of the single parameter in FGDM/IGDM vs. Controls (*p* value < 0.05).

Study Name and Country	Population	Maternal Age (yrs)	GDM Criteria	Measuring Time	Ultrasound System	Main TTE and STE Findings in FGDM/IGDM vs. Controls
Liu F. et al., 2012, China [12]	FGDM = 32 Controls = 60	FGDM = 29.5 Controls = 27.2	OGTT	At 32.2 weeks’ gestation	Siemens	↔ LVEF ↓ LV-GLS
Wang H. et al., 2015, China [13]	FGDM = 35 Controls = 60	FGDM = 30.4 Controls = 30.7	ADA	At 33.5 weeks’ gestation	GE	↑ IVS thickness ↔ LVEF ↔ LV-GLS ↓ RV-FWLS
Kulkarni A. et al., 2017, USA [14]	FGDM = 82 Controls = 70	FGDM = 33 Controls = 31	OGTT	At 25.3 weeks’ gestation	GE	↑ IVS thickness ↔ E/A ratio, LV-MPI ↓ LVEF, LV-GLS, LV-GCS
Miranda J.O. et al., 2018, Portugal [15]	FGDM = 76 Controls = 53	FGDM = 33 Controls = 32	OGTT	At 31.2 weeks’ gestation	GE	↑ IVS thickness ↔ MAPSE, LV-MPI, LV-GLS ↓ RV-GLS
Rolf N. et al., 2018, Germany [16]	FGDM = 53 Controls = 127	NR	IADPSG	At 30.7 weeks’ gestation	Philips	↓ LV-GLS, RV-FWLS ↑ inter- and intra-ventricular dyssynchrony
Aguilera J. et al., 2020, UK [17]	FGDM = 161 Controls = 483	FGDM = 34.5 Controls = 32.4	IADPSG	At 36 weeks’ gestation	Canon	↔ E/A ratio, E/e’ ratio ↔ LV-MPI, TAPSE ↓ LVEF, LV-GLS, RV-GLS
Lee-Tannock A. et al., 2018, Australia [18]	FGDM = 50 Controls = 126	FGDM = 32 Controls = 32	OGTT	At 38.2 weeks’ gestation	Siemens	↔ MAPSE, TAPSE, E/A ratio ↔ LV-GLS ↓ RV-GLS
Wang D. et al., 2021, China [19]	FGDM = 58 Controls = 58	FGDM = 32.3 Controls = 30.8	OGTT	At 28 weeks’ gestation	GE	↓ LV-FAC, RV-FAC ↓ LV-GLS, RV-GLS
Yovera L. et al., 2021, UK and Spain [20]	FGDM = 69 Controls = 153	FGDM = 34.1 Controls = 32	OGTT	At 33.5 weeks’ gestation	Philips	↔ LV-GLS ↓ RV-FAC, TAPSE, RV-GLS
Song Y. et al., 2021, China [21]	FGDM = 60 Controls = 60	FGDM = 31.6 Controls = 31.3	OGTT	At 29.6 weeks’ gestation	GE	↑ IVS thickness ↔ LV-GLS ↓ RV-FAC, RV-GLS
Gireadă R. et al., 2022, Romania [22]	FGDM = 33 Controls = 30	FGDM = 31.3 Controls = 30	OGTT	At 33.4 weeks’ gestation	GE	↓ RV-FS, RV-FAC ↔ LV-GLS, RV-GLS
Huang P. et al., 2023, China [23]	FGDM = 49 Controls = 50	FGDM = 32 Controls = 30	OGTT	At 28.1 weeks’ gestation	GE	↓ LVEF, LV-FAC, RV-FAC ↓ LV-GLS, RV-GLS
Al-Biltagi M. et al., 2015, Egypt [24]	IGDM = 25 Controls = 45	IGDM = 28.6 Controls = 27	OGTT	At 38.7 weeks’ gestation	GE	↓ E/A ratio, LV-FS, ↓ LV-GLS ↓ RV-TEI index
Cade W.T. et al., 2017, USA [25]	IGDM = 25 Controls = 23	IGDM = 30 Controls = 23	OGTT	At 37 weeks’ gestation	GE	↔ RWT, LV mass, LVEF ↓ LV-GLS, RV-GLS
Iwashima S. et al., 2019, Japan [26]	IGDM = 36 Controls = 39	IGDM = 33 Controls = 31	OGTT	At 39 weeks’ gestation	GE	↔ E/A ratio, LVEF ↑ endocardial GCS ↔ LV-GLS
Smith A. et al., 2021, Ireland [27]	IGDM = 40 Controls = 40	IGDM = 35 Controls = 34	OGTT	At 39 weeks’ gestation	GE	↑ IVS thickness ↑ LV length ↓ LV-GLS, RV-FWLS
Samanth J. et al., 2021, India [28]	IGDM = 132 Controls = 66	NR	OGTT	At 36.6 weeks’ gestation	GE	↑ IVS thickness, LV mass ↔ LVEF, LV-FS ↓ LV-GLS, RV-GLS
Sonaglioni et al., 2022, Italy [29]	IGDM = 30 Controls = 30	IGDM = 34.1 Controls = 33.9	OGTT	At 36.2 weeks’ gestation	Philips	↑ IVS thickness, LV mass, E/e’ ratio ↔ MAPSE, LVEF, TAPSE ↓ LV-GLS, LV-GCS, RV-GLS

**Table 2:** Conventional and functional echocardiographic parameters measured in FGDM/IGDM and controls by the included studies. Data are expressed as the median and interquartile range. EDD, end-diastolic diameter; FGDM, fetuses of gestational diabetic mothers; FS, fractional shortening; GCS, global circumferential strain; GLS, global longitudinal strain; IGDM, infants of gestational diabetic mothers; IVS, interventricular septum; LV, left ventricular; LVEF, left ventricular ejection fraction; LVMi, left ventricular mass index; MAPSE, mitral annular plane 4systolic excursion; MPI, myocardial performance index; NS, not statistically significant; RV, right ventricular; RVIT, right ventricular inflow tract; RWT, relative wall thickness; TAPSE, tricuspid annular plane systolic excursion.

Echocardiographic Parameters	Number of Studies for Parameters Assessed (%)	Sample Size FGDM/IGDM vs. Controls	FGDM/IGDM	Controls	*p* Value
IVS thickness (mm)	9 (50.0)	541 vs. 544	4.1 (2.2–7.2)	3.3 (1.9–5.1)	<0.05
LV-EDD (mm)	3 (16.6)	99 vs. 99	15.9 (13.9–17.3)	16.1 (13.9–18)	NS
RWT	2 (11.1)	55 vs. 53	0.37 (0.35–0.38)	0.32 (0.3–0.33)	<0.05
LVMi (g/m^2^)	2 (11.1)	55 vs. 53	43.0 (36.6–49.5)	38.1 (29.9–46.3)	<0.05
E/A ratio	7 (38.8)	484 vs. 867	0.77 (0.56–0.92)	0.81 (0.65–1.26)	<0.05
E/e’ ratio	3 (16.6)	323 vs. 579	10.4 (9.3–11.5)	7.9 (6.1–9.3)	<0.05
LV-FS (%)	5 (27.7)	291 vs. 217	37.4 (35–39.2)	38.4 (34–41.6)	NS
LV-FAC (%)	3 (16.6)	140 vs. 138	41.9 (35.3–45.3)	46.2 (42.4–49)	<0.05
LVEF (%)	10 (55.5)	615 vs. 911	64.9 (58–71.6)	66.6 (61.6–72)	<0.05
LV-SV (mL)	4 (22.2)	188 vs. 163	2.1 (0.7–4)	2.4 (0.7–4.4)	<0.05
MAPSE (mm)	4 (22.2)	216 vs. 269	5.3 (4.2–6.7)	5.6 (4.5–7)	<0.05
LV-MPI	4 (22.2)	379 vs. 666	0.53 (0.46–0.6)	0.51 (0.37–0.58)	NS
LV-GCS (%)	3 (16.6)	148 vs. 130	−21.2 (19.9–22.6)	−23.2 (17–26.3)	<0.05
LV-GLS (%)	18 (100.0)	1046 vs. 1573	−18.8 (11.6–24.2)	−21.5 (11.8–28)	<0.05
RVIT (mm)	3 (16.6)	103 vs. 100	13.5 (9.9–16.5)	13.3 (10.2–15.5)	NS
TAPSE (mm)	5 (27.7)	377 vs. 752	7.7 (6.2–9)	7.9 (6.7–9.2)	<0.05
RV-Tei index	2 (11.1)	157 vs. 111	0.48 (0.4–0.56)	0.32 (0.28–0.36)	<0.05
RV-FAC (%)	4 (22.2)	209 vs. 291	34.5 (30.9–38)	40.4 (36.8–43.5)	<0.05
RV-GLS (%)	14 (77.7)	871 vs. 1329	−19.7 (13.7–26.6)	−22.4 (15.5–32.6)	<0.05

**Table 3:** Results of meta-regression analysis of GDM effect on LV-GLS. BMI, body mass index; CI, confidence interval; FGDM, fetuses of gestational diabetic mothers; GDM, gestational diabetes mellitus; GE, General Electric; GLS, global longitudinal strain; HbA1C, glycosylated hemoglobin; IGDM, infants of gestational diabetic mothers; LV, left ventricular; OGTT, oral glucose tolerance test.

Moderators	Coefficient	Standard Error	95%CI Lower	95%CI Upper	*p*-Value
Gestational age (weeks)	0.1639	0.093	−0.0184	0.3462	0.08
Maternal age (yrs)	−0.0375	0.1513	−0.334	0.2591	0.80
Maternal BMI (kg/m^2^)	0.0144	0.0738	−0.1303	0.1591	0.84
Maternal HbA1C (%)	0.9653	0.8616	−0.7234	2.6539	0.26
White ethnicity	−0.5056	0.7857	−2.0456	1.0344	0.52
GDM criteria: OGTT	0.207	0.7537	−1.2702	1.6842	0.78
Ultrasound system: non-GE	0.5616	0.5326	−0.4822	1.6054	0.29
Frame rate (fps)	0.0135	0.0079	−0.0019	0.0289	0.09
IGDM/FGDM heart rate (bpm)	−0.0057	0.0549	−0.1133	0.1019	0.92
No anti-diabetic therapy	−0.6296	0.7445	−2.0888	0.8296	0.40

**Table 4:** Results of meta-regression analysis of GDM effect on RV-GLS. BMI, body mass index; CI, confidence interval; FGDM, fetuses of gestational diabetic mothers; GDM, gestational diabetes mellitus; GE, General Electric; GLS, global longitudinal strain; HbA1C, glycosylated hemoglobin; IGDM, infants of gestational diabetic mothers; OGTT, oral glucose tolerance test; RV, right ventricular.

Moderators	Coefficient	Standard Error	95%CI Lower	95%CI Upper	*p*-Value
Gestational age (weeks)	0.0814	0.121	−0.1558	0.3187	0.50
Maternal age (yrs)	−0.2403	0.2636	−0.757	0.2764	0.36
Maternal BMI (Kg/m^2^)	0.0347	0.0555	−0.074	0.1434	0.53
Maternal HbA1C (%)	−1.984	1.792	−5.4962	1.5283	0.27
White ethnicity	1.3748	0.9065	−0.4019	3.1515	0.13
GDM criteria: OGTT	0.9596	1.8151	−2.5979	4.5171	0.60
Ultrasound system: non-GE	0.5668	1.4947	−2.3628	3.4964	0.70
Frame rate (fps)	0.005	0.0108	−0.0161	0.0261	0.64
FGDM/IGDM heart rate (bpm)	−0.0746	0.0641	−0.2002	0.051	0.24
No anti-diabetic therapy	0.1579	0.473	−0.7692	1.0849	0.73

## Data Availability

Data extracted from included studies will be publicly available on Zenodo (https://zenodo.org).
